# Do we really need a new definition of dyslexia? A commentary

**DOI:** 10.1007/s11881-024-00305-y

**Published:** 2024-03-25

**Authors:** Maggie Snowling, Charles Hulme

**Affiliations:** 1 University of Oxford and St. John’s College, Oxford, England; 2 Oxford Brookes University, Oxford, England

**Keywords:** Dyslexia, Definition, Dimensional disorder, Language learning difficulty

## Abstract

We provide a commentary on current debates about the definition of dyslexia. We agree with others that dyslexia is best thought of as a dimensional disorder with the best established causal risk factor being a deficit in phonological processing. Dyslexia is particularly common in children from families with a history of dyslexia and in children with preschool language difficulties. We argue that definitions may differ depending upon their purpose. Traditional discrepancy definitions may be useful for research purposes, but when considering the provision of educational services discrepancy definitions are not useful since all children with reading difficulties require reading intervention regardless of their level of IQ.

The nature and definition of dyslexia have been debated ever since it was first identified at the close of the nineteenth century (Kirby & Snowling, 2022). Among the early pioneers, perhaps, it was Samuel Orton’s description of the condition that is closest to current understanding. Although Orton is usually remembered for his attempt to explain the ‘twisting of symbols’ observed in the reading and writing of children with dyslexia, he also documented the familial nature of the disorder, and the tendency for family members to show deficits not necessarily in reading or writing but frequently in speech and language. Here, we will reflect on the points raised by Catts and Petscher and Wolf and colleagues and propose that it is critical for a definition of dyslexia to be evidence-based, and useful in guiding research and practice. Our commentary draws on our longitudinal studies of reading and provides a UK perspective on definitions of dyslexia.

In our view, the broad form of a definition of dyslexia is clear and needs little by way of revision. We believe the core of the definition proposed by the UK Rose Review remains useful and accurate (Rose, 2009).

“Dyslexia is a learning difficulty that primarily affects the skills involved in accurate and fluent word reading and spelling. Characteristic features of dyslexia are difficulties in phonological awareness, verbal memory and verbal processing speed. Dyslexia occurs across the range of intellectual abilities. It is best thought of as a continuum, not a distinct category, and there are no clear cut-off points”.

The key points here are:

- 1. Dyslexia is a difficulty in learning to decode/encode print.
- 2. It is associated with phonological problems.
- 3. It may occur at different levels of ability.
- 4. Dyslexia is a dimensional disorder—where we set a cut-off for identification that is to some extent arbitrary.

In addition, Rose noted that an indication of the severity of dyslexia may be provided by how an individual responds to intervention and that dyslexia often co-occurs with other disorders although these are not themselves markers of dyslexia. It is important not to lose sight of these basic facts about dyslexia, but here, we will reflect on some detailed nuances where debates still continue.

Before getting into details about current areas of detailed debate, it is probably worth making three general points.

## A dimensional disorder

First, the definition of dyslexia as a dimensional disorder sometimes causes concern. To put it bluntly, if there is no universally accepted cut-off, does this make the disorder less real? We would argue that dimensional disorders are common in medicine: Hypertension (high blood pressure) is a good example. The World Health Organization (WHO) defines hypertension as when “the systolic blood pressure readings on two different days is ≥ 140 mmHg and/or the diastolic blood pressure readings on both days is ≥ 90 mmHg”. Hypertension is a very real problem and can be effectively treated with medications resulting in the prevention of many premature deaths and other medical problems. Dyslexia arguably is the same—it can be detected, and it can be effectively treated, but the cut-off in terms of a reading score needed for a diagnosis is to some extent arbitrary, just as in the case of hypertension. A patient with systolic blood pressure readings on two different days of 139 mmHg is not qualitatively different from a person with systolic blood pressure of 141 mmHg. It is just that 140 mmHg is a boundary above which there is a consensus among many in the medical profession that the advantages of medication outweigh the possible side effects.

## Dyslexia and general ability (IQ)

The relevance of measures of general ability (IQ) to the definition of dyslexia continues to be controversial. Early definitions used a discrepancy approach whereby dyslexia was defined as a problem with reading that, from a regression model, was out of line with a person’s age and IQ (for an example, see Rutter & Yule, 1975). Most contemporary definitions have explicitly moved away from a discrepancy definition.

In our view, the way a disorder is defined may vary according to the purposes the definition is to be used for. The Rose Review dealt with the educational policy implications of dyslexia, and for this reason, Rose did not adopt a discrepancy definition. A discrepancy definition might for example deem a child with a reading standard score of 85 and an IQ of 110 as worthy of reading intervention, while a child with a reading standard score of 85 and an IQ of 99 might be deemed not to require intervention. Such a clinical judgement is dubious, as both children have the same level of reading ability and would be likely to benefit from help to improve their reading. Furthermore, there is little evidence that, across a wide range, IQ is predictive of response to reading instruction (Hatcher, 2000; Hatcher & Hulme, 1999; Stuebing et al., 2009).

In contrast to the use of a dyslexia definition to guide treatment, a discrepancy definition may work well in a research context for identifying children with “unexpected” reading problems. Traditionally, many research studies of dyslexia have selected children based on a discrepancy between reading level and age and IQ. There is no doubt that IQ in the general population correlates with reading ability (Rutter & Yule, 1975); hence, using a discrepancy definition in research studies will exclude children whose reading problems are associated with more general learning difficulties (a child with a reading standard score of 85 and an IQ of 85 may be considered to have a less specific difficulty with reading, than a child with the same reading standard score, but an IQ of 110). While it remains unclear the extent to which such selection criteria bias our understanding of the typical characteristics of children with reading problems, it should be noted that individuals of lower measured IQ may well be those with co-occurring conditions, such as developmental language or coordination disorder (Snowling et al., 2020a, 2020b).

## Should the causes of a disorder be a part of its definition?

When we talk about the causes of a developmental disorder, we are talking about causal risk factors (Hulme & Snowling, 2009). The development of a disorder typically reflects the interaction of many different causal factors that can be identified at different levels (cognitive, environmental, and biological risk factors). Dyslexia is a highly heritable condition, with the genes involved operating to influence the development of a left hemisphere reading “circuit” involving occipital, temporal, and frontal brain regions. At a cognitive level, the best-established cause of reading problems is a deficit in processing phonological (speech sound) information.

We doubt that these causal risk factors should be made a part of the definition of dyslexia. While we agree that dyslexia has a neurobiological basis, and one of the strongest known risk factors is having a first-degree relative with reading difficulties (Snowling & Melby-Lervåg, 2016) suggesting its origins are likely to be genetic, it is not feasible currently to use biological markers for its identification. Indeed, although numerous genetic variants have been found to be associated with the ‘dyslexia phenotypes’, individual genes have tiny effect sizes accounting for very little variance in reading in the population (Doust et al., 2022). Recent studies that use polygenic risk scores (summing the influence of all identified relevant genes) show that genetic variation accounts for at most 12–16% of the variance in educational attainment (years of education measured in a sample of some 3 million participants; Okbay et al., 2022). Moreover, their effects are not direct and include effects of gene-environment correlation and assortative mating (Meyer et al., 2023 for a review).

Similarly, structural and functional neuroimaging methods, which are costly, do not yet provide information that would be useful in making individual diagnoses of ‘dyslexia liability’. In contrast, measures of cognition and behaviour are psychometrically reliable and of low cost and directly assess the factors that may form the targets of intervention (reading, spelling, and phonological skills). For these reasons, we do not see the rationale for including in a definition of dyslexia a reference to its likely neurobiological causes.

## Toward a definition of dyslexia?

### The core deficit in phonological processing

The strong relationship between phonological skills and reading and the role of phonological deficits in dyslexia are well known (Melby-Lervåg, Lyster & Hulme, 2012). There is also evidence that phonological deficits are observed in children who go on to be dyslexic prior to reading instruction (Snowling et al., 2019); it has also been shown that phonological skills predict individual differences in reading fluency across alphabetic orthographies (Caravolas et al., 2013). Moreover, while there is relatively strong evidence that phoneme awareness and letter knowledge are causally related to reading problems (e.g. Hulme et al., 2012), rapid improvements in these skills during the first year of formal schooling appear to be strongly predicted by earlier variations in oral language that can be measured in the preschool years (Hulme et al., 2015). Thus, perhaps surprisingly, oral language skills appear to have a direct role in laying the foundations for the development of decoding skills (via effects on the development of phoneme awareness and letter-sound knowledge) as well as being critical for the development of reading comprehension. It seems to us important therefore to acknowledge early oral language difficulties as an important risk factor for dyslexia.

The question remains, however, as to whether our understanding of the causal risk factors for a disorder should be a part of its definition. We believe that it is better not to include claims about causal risk factors in the definition of dyslexia. As referenced above, the WHO definition of hypertension does not reference the numerous causal risk factors operating (genetics, diet, exercise). Similarly, the WHO’s definition of obesity does not mention causal risk factors “obesity … abnormal or excessive fat accumulation that presents a risk to health … a body mass index (BMI) over 30 is obese”.

In our view, there may be advantages in following these examples from medicine and defining dyslexia in terms of what we can easily measure directly at the behavioural level (reading and spelling). While it remains essential to understand the causal risk factors operating in dyslexia, we are not convinced that these should be included in its definition; rather, we would follow other classification systems such as DSM5 (American Psychiatric Association, 2013) and ICD11 (World Health Organisation, 2018), as well as the UK’s Rose (2009) definition and focus on cognitive and behavioural symptoms rather than causal risk factors.

### The multifactorial basis of dyslexia

Like many, we have been influenced by the multifactorial model of Pennington and colleagues (Pennington, 2006; McGrath, Petersen & Pennington, 2020). However, there is a danger of going beyond the data when discussing the possible causal risk factors for dyslexia outside of phonology. Wolf et al. propose three such factors: naming speed, executive function, and visual-orthographic skills. We believe that the evidence for rapid-automatized naming (RAN) as a risk factor for the development of reading fluency is relatively strong (e.g. Lervag & Hulme, 2009). However, we do not know of any good evidence that executive function and visual-orthographic skills are causal risk factors. In fact, we would see deficits in orthographic skills as a consequence of a reading problem rather than a cause.

Catts et al. (2024 - this issue) suggest that difficulties in oral language, visual processing, processing speed, and procedural learning are putative causal risk factors for dyslexia. We agree strongly that oral language deficits that predate reading difficulties are a powerful risk factor for both later decoding and reading comprehension problems. However, we contend that the evidence for visual processing having a causal status is lacking, and we have argued elsewhere (as noted by Catts et al., 2024 - this issue) that we find no evidence for a causal role of procedural learning (West et al., 2021). Another sticky point is what is meant by executive function and processing speed and how does the latter differ from naming speed. Both constructs are considered important but are themselves multi-faceted, and there is a lack of direct evidence for their role in learning to read (Malone et al., 2022). Like ‘protective factors’, these are familiar ideas which could be tested empirically but at present might more properly be considered rather speculative hypotheses.

### Comorbidity

Related to the multiple deficit view of dyslexia is the issue of comorbidity—the co-occurrence of dyslexia with other disorders. Given that comorbidity is the norm in developmental disorders, it would seem important to note, as did Rose (2009), that dyslexia frequently co-occurs with difficulties in attention, language, mathematics, and motor coordination, but ‘these are not features of dyslexia’ per se. Moreover, until there is further research on the shared risk factors between dyslexia and other disorders, as opposed to specific risk factors, we can only conjecture as to how these affect the progression of dyslexia over time—specifically, are they additive or interactive? (Moll et al., 2020). In a similar vein, dyslexia may lead to low confidence, poor self-esteem, and possibly internalizing or externalizing disorders, but the causal connections are unclear (Donolato et al., 2022). We are on safer ground in stating that poor vocabulary or poor reading comprehension can be a downstream effect of dyslexia, though note, that such difficulties are much less marked than those experienced by children with developmental language disorder (Snowling et al., 2020a, 2020b). We would note a point of disagreement with Catts and Petscher (2022) here. We do not believe that including comorbid disorders in the definition of dyslexia is useful. In fact, we would argue that the failure to recognize clearly that comorbid disorders are distinct from dyslexia has led to confusion in this area. To take a clear example, motor difficulties may be frequently comorbid with dyslexia, but motor problems reflect cognitively distinct mechanisms, and most critically interventions to improve motor skills cannot be expected to reduce reading difficulties as was claimed for example by Dore (Rack et al., 2007; Stephenson & Wheldall, 2008).

### Disorder or difference?

A thorny issue is whether dyslexia should be considered a disorder or a disability. This issue is central to the concerns of the neurodiversity movement. In line with the dimensional view, Wolf et al. prefer the notion of a difference. Our views differ from theirs; we prefer to describe dyslexia as a specific learning disorder (affecting the acquisition of reading and spelling skills). While educationists have tended to reject the notion of ‘dyslexia’ as a disorder—believing that this is a medical model of the issues affected individuals face—even medical models of the disease need to determine diagnostic categories if treatment is to be prescribed, as in the case of hypertension discussed earlier. So even though reading skills are normally distributed in the population, we argue that rejection of a category for identification increases the probability that ‘cases’ needing intervention will be missed, and less principled criteria will be used in identification—resources may end up directed to those who obtain private assessments or are from certain social classes or ethnic groups. For similar reasons, we see it as important to prioritize needs based on core characteristics; otherwise, it will be difficult to know how and when to provide intervention. That is not to say that intervention should only be provided for those who fulfil definitional criteria for dyslexia. As our recent Delphi study has shown (Carroll et al., n.d.), there is consensus among experts that many individuals with poor literacy skills deserve to be offered intervention irrespective of whether they fulfil strict diagnostic criteria for ‘dyslexia’.

## Conclusions

Although scientific understanding of dyslexia is advanced, its definition continues to be debated. As we have outlined, the core of a definition of dyslexia seems to us to be clear and relatively widely accepted. We have discussed some of the nuances that surround the definition, but these in our view are details rather than fundamental issues. Reading skills in the population are normally distributed, and dyslexia is a term that is used to refer to the bottom end of the distribution (typically between 5 and 10% of the population). We favour using measures of behaviour (reading and spelling skills) as the primary means of diagnosing dyslexia. Although there is a degree of arbitrariness in where the “cut-off” for dyslexia is placed, we do believe that the use of dyslexia as a categorical label is useful both in educational practice and research. The dimensional view clearly entails, however, that such a category may be modified by adjectives—a person might be described as mildly or severely dyslexic depending on their level of literacy needs. It is important in relation to educational policy that it is recognized that reading difficulties are both common and remediable and that suitable interventions are made widely available. Dyslexia is persistent although its effects will differ according to context and the individual differences in related skills that the child brings to the task of learning (see Carroll et al., for a consensus study; Catts & Petscher, 2022). Moreover, there is a good understanding of some of the causal risk factors associated with dyslexia and a significant evidence base of effective interventions for reading and language difficulties. The issues involved in scaling up methods of screening and identification and, following these, providing evidence-based interventions need to be at the forefront of policy and practice (Newbury et al., 2022).
